# Optical-Resolution Photoacoustic Microscopy Using Transparent Ultrasound Transducer

**DOI:** 10.3390/s19245470

**Published:** 2019-12-11

**Authors:** Haoyang Chen, Sumit Agrawal, Ajay Dangi, Christopher Wible, Mohamed Osman, Lidya Abune, Huizhen Jia, Randall Rossi, Yong Wang, Sri-Rajasekhar Kothapalli

**Affiliations:** 1 Department of Biomedical Engineering, The Pennsylvania State University, State College, PA 16802, USA; haoyangchen@psu.edu (H.C.); sua347@psu.edu (S.A.); axd571@psu.edu (A.D.); clw5710@psu.edu (C.W.); mqo5163@psu.edu (M.O.); lua28@psu.edu (L.A.); huj202@psu.edu (H.J.); yxw30@psu.edu (Y.W.); 2 Huck Institutes of the Life Sciences, The Pennsylvania State University, State College, PA 16802, USA; rmr29@psu.edu; 3 Penn State Cancer Institute, The Pennsylvania State University, Hershey, PA 17033, USA; 4 Graduate Program in Acoustics, The Pennsylvania State University, State College, PA 16802, USA

**Keywords:** photoacoustic microscopy, ultrasonic transducer, optical-resolution photoacoustic microscopy, transparent ultrasound transducer, ultrasound stimulation

## Abstract

The opacity of conventional ultrasound transducers can impede the miniaturization and workflow of current photoacoustic systems. In particular, optical-resolution photoacoustic microscopy (OR-PAM) requires the coaxial alignment of optical illumination and acoustic-detection paths through complex beam combiners and a thick coupling medium. To overcome these hurdles, we developed a novel OR-PAM method on the basis of our recently reported transparent lithium niobate (LiNbO_3_) ultrasound transducer (Dangi et al., Optics Letters, 2019), which was centered at 13 MHz ultrasound frequency with 60% photoacoustic bandwidth. To test the feasibility of wearable OR-PAM, optical-only raster scanning of focused light through a transducer was performed while the transducer was fixed above the imaging subject. Imaging experiments on resolution targets and carbon fibers demonstrated a lateral resolution of 8.5 µm. Further, we demonstrated vasculature mapping using chicken embryos and melanoma depth profiling using tissue phantoms. In conclusion, the proposed OR-PAM system using a low-cost transparent LiNbO_3_ window transducer has a promising future in wearable and high-throughput imaging applications, e.g., integration with conventional optical microscopy to enable a multimodal microscopy platform capable of ultrasound stimulation.

## 1. Introduction

Optical-resolution photoacoustic microscopy (OR-PAM) has recently gained significant attention from the biomedical-imaging community as it provides labelfree optical contrast from physiologically relevant tissue chromophores that are located a few millimeters deep, with subcellular spatial resolution [1,2,3,4,5]. In OR-PAM, a tightly focused laser pulse illuminates the tissue and generates wideband acoustic waves from light-absorbing chromophores that are then detected by an acoustic transducer. Time-resolved photoacoustic waves, in combination with the two-dimensional raster scanning along the *x*-*y* plane (lateral dimension), generate three-dimensional data from which maximum amplitude projection (MAP) and volume-rendered photoacoustic images can be created.

Conventional OR-PAM setups use complex imaging geometries to coaxially align optical illumination and acoustic detection paths. In early OR-PAM setups [6], coaxial alignment was achieved using an acoustic-optic prism combiner consisting of one right-angle prism and one rhomboid prism pressed tightly to a thin layer of silicone oil. The laser light was focused by a system of optical lenses and then passed through the prism combiner before irradiating the tissue. A correction lens was attached to the prism combiner to refocus the light that was defocused through the combiner. Tissue-generated photoacoustic waves propagated through the rhomboid prism and were reflected by the silicone oil layer into the ultrasound detector attached to the prism. Since the entire imaging head, consisting of the above acoustic-optic prism combiner, the transducer, and the focused light, was moved to scan the subject, these systems exhibited slow acquisition speed, limited field of view (FOV), and significant acoustic loss.

The current generation of OR-PAMs reflect the light, instead of the acoustic waves, by sandwiching an aluminium foil in the acoustic-optic combiner. This allows dual axis optic only scanning using a two dimensional galvo mirror to improve the image acquisition speed and generate a wide FOV [7]. The entire imaging head, including the galvo mirror, is submerged in a large volume (70 × 40 × 20 mm^3^) of a nonconducting liquid coupling medium that rests above the imaged subject. Such a bulky imaging head limits high throughput and wearable imaging applications because it constrains animal imaging performed under anesthesia and causes discomfort to living subjects. Moreover, acoustic loss here is still significant because acoustic waves travel through the large coupling medium and the prism combiner before being detected by the transducer. 

Alternatively, some OR-PAMs include a ring-shaped single-element ultrasound transducer to eliminate the off-axis alignment problems of optical illumination and acoustic detection. The focused light is directly delivered through a hole at the center of the transducer, or coupled using a single-mode fiber integrated with a gradient-index (GRIN) lens. The imaging head is then two-dimensionally raster-scanned using mechanical stages to generate volumetric images [8,9,10,11,12]. Although the imaging head is miniaturized in these OR-PAM systems, the FOV, numerical aperture, and imaging speed (due to physical scanning of the imaging head) are still limited. Besides, they still require a-few-milimeter thick water coupling medium above the imaged subject due to long working distances.

The above drawbacks of conventional OR-PAM systems can be addressed if ultrasound detectors are transparent to light. To achieve this, all-optical ultrasound detection technologies, such as Fabry–Pérot etalons [13], microring resonators [14], and other photonic integrated circuits [15] were studied for PAM. Although these are transparent technologies offering high photoacoustic sensitivity, they require complex fiber integration with an additional laser source and other optical-detection instruments. More importantly, they lack ultrasound excitation capabilities for applications that require combined ultrasound sensing and ultrasound tissue stimulation [16,17]. Recently, transparent capacitive micromachined ultrasonic transducers (CMUTs) were developed [18,19]. However, CMUTs need specialized front-end application-specific integrated circuits (ASICs), and involve a complicated fabrication process inside a cleanroom.

In order to address all the above limitations, we recently reported a photoacoustic-imaging technique using an optically transparent bulk piezoelectric lithium niobate (LiNbO_3_) ultrasound transducer [20]. LiNbO_3_ offers several advantages over other piezoelectric materials:

1. It exhibits a good electromechanical coupling coefficient (53%);
2. It demonstrates a high Curie temperature (>1100 °C), making it easy to process through high-temperature sputtering without losing poling [21];
3. It shows promising results in high-frequency ultrasound and PAM applications [22,23,24].

On the basis of these advantages, our previous work [20] introduced two transparent photoacoustic-imaging schematics, one that directly integrated a multimode optical fiber with the transparent LiNbO_3_ transducer, and the other that involved the optical-only scanning of the laser spot over a 10 × 10 mm^2^ LiNbO_3_ window transducer. 

Extending the window-transducer approach, here we present a novel OR-PAM that allows the optical-only scanning of a tightly focused light beam through a 10 × 10 mm^2^ single-element LiNbO_3_ transparent-ultrasound-transducer (TUT) window and demonstrates its applicability to image biological samples. The spatial resolution and signal-to-noise ratio (SNR) of the OR-PAM system were characterized using imaging experiments on resolution test targets and carbon-fiber phantoms. The biological imaging capabilities of the OR-PAM were studied using *ex ovo* chick-embryo chorioallantoic-membrane (CAM) vasculature and imaging melanoma phantoms through a piece of mouse skin. 

The proposed TUT-based OR-PAM approach simplifies the coaxial alignment of optic and acoustic paths without the need for additional optical components (such as acoustic-optic prism combiners and correction lenses) and a large acoustic-coupling medium. Our approach provides other advantages:

1. The TUT can be fixed onto the imaging object (such as the skull of a mouse) to facilitate wearable imaging without a thick coupling medium. In the future, this will likely help in imaging the brains of freely behaving or awake mice in combination with ultrasound stimulation;
2. Depending on the size of the TUT, it enables the high-speed scanning of large areas with single-channel data acquisition.
3. In the future, our TUT approach can also be integrated into conventional optical microscopes to realize a multimodal microscopy platform with ultrasound-stimulation capabilities.

The rest of this paper is organized as follows. Section 2 describes the process of TUT fabrication and a schematic representation of the OR-PAM setup. TUT characterization and validation studies, including carbon-fiber imaging, CAM vasculature, and melanoma depth profiling, are presented in Section 3. The advantages of the proposed TUT-based OR-PAM system, the limitations, and its future directions are discussed in Section 4.

## 2. Materials and Methods

### 2.1. Transducer-Fabrication Processes

As reported in our recent work [20], a 250 µm thick Y-cut 36° LiNbO_3_ wafer was sputtered with 200 nm thick indium tin oxide (ITO) on both sides and then diced into square pieces of 10 × 10 mm^2^, which resulted in ~80% optical transparency in the visible and near-infrared optical-wavelength regions. Square tubing (0.5 in. width, 0.032 in. wall thickness, and 10 mm height) was used as a conductive housing. The bottom electrode and brass tubing were connected using a conductive silver epoxy (E-solder 3022, Von Roll Isola Inc., New Haven, CT, USA) that outlined the 4 edges of the bottom electrode. The conductive silver epoxy had 1 mm thickness and 2 mm total width, with 1 mm covering the bottom electrode. This resulted in an FOV of 9 × 9 mm^2^. The conductive epoxy also acted as an absorber for the surface-acoustic waves generated by the LiNbO_3_ in response to pulsed-light incidence. 

The top electrode was connected to an SMA to BNC connector using a microstranded wire. A nonconducting and transparent epoxy (Epotek-301, Epoxy Technologies Inc., Billerica, MA, USA) was poured until it filled the brass housing. This epoxy was used as the backing layer that would reduce the ringing effect by absorbing vibrational energy, and improve bandwidth [25]. Extra care was taken to ensure that no particles were trapped in the epoxy that may have diffracted the light or caused a shadowing effect. 

**Figure 1:**
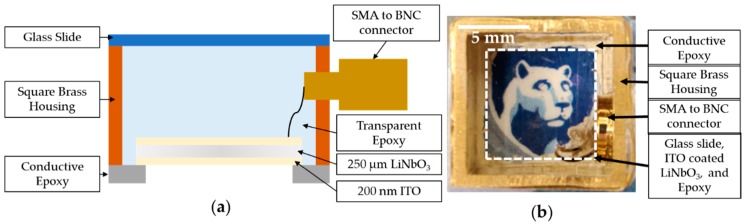
Transparent-ultrasound-transducer (TUT) design based on piezoelectric lithium niobate (LiNbO_3_) material. (**a**) Schematic cross-sectional view of fabricated TUT, which had 10 mm height and 9 × 9 mm^2^ field of view. ITO: Indium tin oxide; (**b**) Photograph of fabricated TUT clearly showing Nittany Lion mascot underneath.

The epoxy is known to shrink during the curing process that can lead to a curved surface inside the brass housing. This curvature can lead to light diffractions and aberrations. In order to ameliorate this effect, a microglass slide with 150 µm thickness was placed on top of the transducer to form a flat surface. A cross-sectional schematic view of the TUT is shown in Figure 1a. Figure 1b shows a photograph of the TUT on top of a Nittany Lion mascot.

### 2.2. Optical-Resolution Photoacoustic-Microscopy Experiment Setup

**Figure 2:**
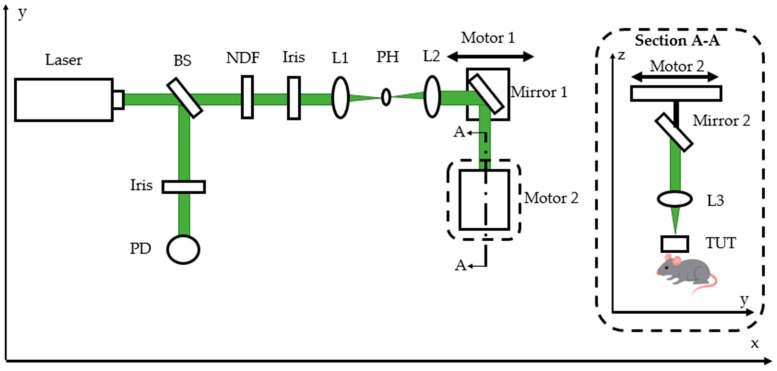
Top-down view schematic of optical-resolution photoacoustic-microscopy (OR-PAM) setup. Raster scanning achieved by Motor 1 moving Mirror 1 to perform *x*-axis scan. Motor 2 moved Mirror 2 and L3 to perform *y*-axis scan. BS: beam sampler; NDF: neutral density filter; PD: photodiode; PH: pinhole; L1, L2, L3: planoconvex lenses with 50, 75, and 50 mm focal lengths, respectively.

A schematic representing the top-down view of the OR-PAM setup is shown in Figure 2. The system employed a high-speed pulsed laser (GLPM-10, IPG Photonics; 532 nm wavelength; 1.4 ns pulse duration; tunable pulse-repetition rate in the range of 10–600 kHz; tunable pulse energy between 1.6 and 19 µJ). The 4 mm diameter laser beam passed through a beam sampler (BSF10-A, Thorlabs Inc., Newton, NJ, USA) that diverted 10% of its energy to a photodiode (DET10A, Thorlabs Inc.) used to synchronize with a high-speed (1 gigasample per second) 16 bit data-acquisition system (Razormax-16, Dynamic Signals LLC, Lockport, IL, USA) connected to a computer. 

The remaining 90% of the beam energy passed through a neutral density filter (NDC-50C-4M, Thorlabs Inc., Newton, NJ, USA) and an iris before entering a spatial filter system. A 20 µm pinhole (P20D, Thorlabs Inc., Newton, NJ, USA) and 2 lenses, with focal lengths 50 (LA1131-A, Thorlabs Inc., Newton, NJ, USA) and 75 mm (LA1608, Thorlabs Inc., Newton, NJ, USA) respectively, were then used to filter and collimate the beam. In order to raster-scan the sample for imaging, 2 motorized stages (NRT-1000, Thorlabs Inc., Newton, NJ, USA) were used to guide the light along the *x* and *y* axes for scanning (Figure 2). A 45° mirror was mounted onto Motor-1, which moved along the *x*-axis. Motor-2 was mounted on a vertical stage (on the *z*-axis) and drove another 45° mirror and a 50 mm focal-length planoconvex lens (LA1131-A, Thorlabs Inc., Newton, NJ, USA) along the *y*-axis. The focused light passed through the TUT mounted just above the imaging sample. A thin layer (~1 mm) of deionized water was used as a coupling medium to receive photoacoustic waves generated from the tissue.

## 3. Results and Discussion

### 3.1. System and Transducer Characterization

**Figure 3:**
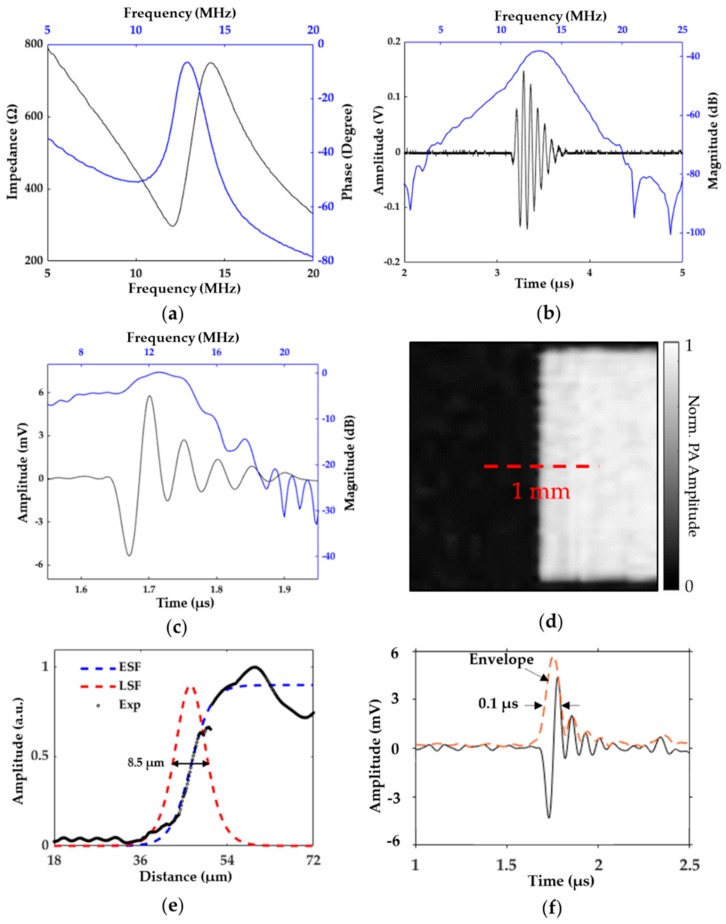
Results of transparent lithium niobate ultrasound-transducer-based OR-PAM system characterization. (**a**) Measured electrical impedance results; (**b**) Pulse-echo response; (**c**) Photoacoustic pulse response of USAF resolution test target; (**d**) Maximum-amplitude-projection (MAP) image of target via edge scanning. PA: photoacoustic; (**e**) Edge-response data and fitted line-spread-function (LSF) curve showed 8.5 µm lateral resolution. ESF: edge spread function; (**f**) Gaussian enveloped curve fitted profile showed 150 µm axial resolution.

First, the transducer was evaluated by analyzing its electrical impedance using a vector network analyzer (Agilent E5100A, Keysight Technologies, Inc., Santa Rosa, CA, USA). Impedance measurements are used to estimate the effective electromechanical coupling coefficient, k*_eff_*, of the transducer, which represents its efficiency to convert between electrical and mechanical energy [26]. As seen in Figure 3a, the resonance and antiresonance frequencies were measured to be 12.05 and 14.22 MHz, respectively, with a resultant k*_eff_* of ~0.53. 

Next, we performed pulse-echo and hydrophone measurements on the TUT using the methods described in our earlier work [20]. Pulse-echo measurement showed a center frequency of 13 MHz and a fractional bandwidth of 25%, as seen in Figure 3b. The hydrophone measurements showed a peak pressure of 85 kPa at 5.4 mm distance from the transducer surface. Our photoacoustic-pulse response was acquired via a USAF resolution test target (R3L3S1P, Thorlabs Inc., Newton, NJ, USA) and amplified by a preamplifier (Olympus 5073PR, Olympus NDT Inc., Waltham, MA, USA) with 39 dB gain. The laser energy measured by a pyroelectric energy meter (PE9-ES-C, Ophir-Spiricon, LLC, North Logan, UT, USA) after spatial filtering was found to be approximately 250 nJ. As seen in Figure 3c, our received photoacoustic signal was then averaged 100 times, and showed a center frequency of 13 MHz with a −6 dB fractional bandwidth of 60%. The SNR was calculated as the 20 log_10_ ratio of the PA signal amplitude to the standard deviation of noise, which was equal to 38 dB.

Next, the lateral resolution of the OR-PAM system was measured by linear scanning along the edge of a ~2 × 2 mm^2^ square block of the USAF resolution-test target. During this test, the block was scanned with a 0.5 µm step size. Figure 3d shows the MAP image of the square edge, with a dashed line showing the 1 mm long scan length. The experiment data of the edge-spread function are shown in Figure 3e. The line-spread function was obtained by taking the first derivative of the fitted edge-spread function; its full-width half-maximum (FWHM) showed a lateral resolution of 8.5 µm. The axial resolution of the system was then estimated by taking the FWHM of a Gaussian envelope applied to a photoacoustic signal from the target. The FWHM was found to be 0.1 µs, which was equal to 150 µm in water, as seen in Figure 3f.

The axial resolution of the PAM system is inversely proportional to the bandwidth of the acoustic receiver and estimated to be 0.88 c/B [27], where B is the −6 dB bandwidth in MHz and c is the ultrasound velocity inside the tissue medium. Using this relation, the axial resolution of the proposed OR-PAM system was expected to be ~167 µm, which aligned well with the experimentally observed value of 150 µm. This axial resolution could further be improved by increasing the TUT’s bandwidth using a stronger acoustic absorption material as the backing layer. This would also likely improve the SNR and spatial resolution of the OR-PAM system. Additionally, since no matching layer was used in the current TUT, the transmitted acoustic energy propagated through the tissue was ~17%, considering the acoustic impedance of the LiNbO_3_ wafer and the tissue was 34 and 1.5 MRayls, respectively. If a transparent matching layer with proper acoustic impedance was added, such as a two-matching-layer design using glass slide and parylene coating, we could achieve transmission coefficient as high as ~45%, as the acoustic impedance mismatch between the piezoelectric material and the tissue would be reduced. This would increase our acoustic transmission and receiving sensitivities, and further result in an improved SNR.

### 3.2. Phantom- and Biological-Tissue-Imaging Experiments

**Figure 4:**
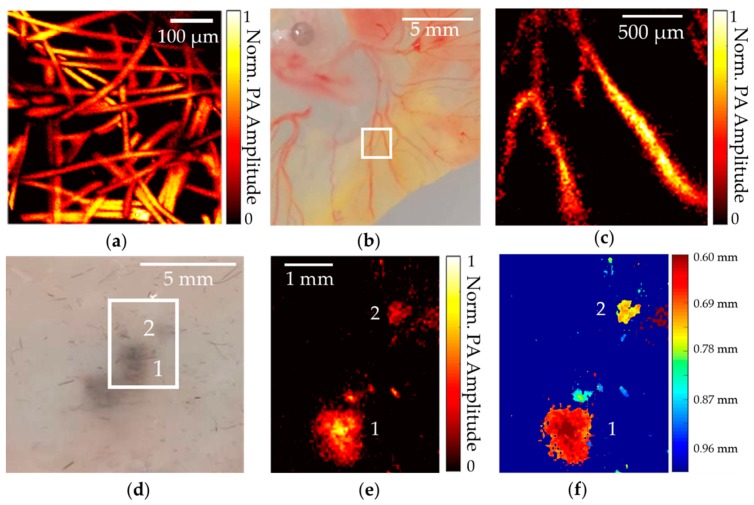
Imaging capabilities of transparent ultrasound-transducer-based OR-PAM system. (**a**) MAP image of carbon-fiber phantom with 0.5 × 0.5 mm^2^ area. PA: photoacoustic; (**b**) Photograph of chick-embryo chorioallantoic membrane (CAM) with imaging area marked by blue box; (**c**) MAP image of CAM vasculature inside blue box of (**b**); (**d**) Photograph of melanoma phantom; (**e**) MAP image of melanin particles detected under mouse skin; (**f**) Color-coded depth profiling of melanoma phantom. Color bar represents depth relative to skin surface.

Next, our OR-PAM system was validated by imaging a 12 µm diameter dense carbon-fiber network (that simulated capillary blood vessels) embedded in an agarose phantom gel. Step size was set at 2 µm to cover an area of 0.5 × 0.5 mm^2^. The photoacoustic signal was then acquired via the high-speed data-acquisition system and averaged 100 times to generate the image. This MAP image of the carbon fiber can be seen in Figure 4a, where each fiber is clearly distinguishable with sufficient resolution and contrast.

Further, we demonstrated the feasibility of utilizing the OR-PAM for vasculature imaging using chicken embryos. Chicken embryos were used as an animal model to visualize different development phases [28], and OR-PAM could reveal their important vasculature information for clinical relevance [29]. 

For this study, Day 4 fertile chicken eggs (E4) were obtained from the Poultry Education and Research Center (PERC) at The Pennsylvania State University. These eggs were gently cracked, and the embryos carefully placed on weigh boats under sterile conditions. The embryos were then incubated at 38 °C with 3% CO_2_ in a humidified incubator. For imaging, the CAM attached to each embryo was removed by cutting around its edges, and then each embryo was quickly and gently transferred to a Petri dish and rinsed with deionized water. Finally, each embryo was placed on top of an agarose-gel phantom bed for imaging. Figure 4b shows a photograph of the CAM: the scanning area is marked by a white box. Scan step size was set at 20 µm to cover a 2 × 2 mm^2^ area, and imaging data were averaged 500 times to provide a sufficient SNR. The vasculature image from MAP, seen in Figure 4c, clearly shows the vascular-branch pattern marked in Figure 4b with adequate contrast and resolution.

Next, the OR-PAM’s application in melanoma imaging was demonstrated by scanning a melanoma phantom. The depth of melanoma invasion under the skin, also known as Breslow’s depth, is one of the three most important prognostic factors in melanoma detection, and it reveals important details about how tumor cells invade [30]. To demonstrate the feasibility of TUT-based OR-PAM wearable imaging of melanoma patients without the need for thick gel coupling, we conducted the following melanoma-tissue experiment. 

Approximately 2 mg of melanin particles (M8631, Sigma Aldrich, St. Louis, MO, USA; optical absorption coefficient ~1100 cm^−1^ at 532 nm [31]) was mixed with 100 mg of 1.5% agarose phantom gel and placed under a piece of mouse skin at different depths. The scan area was set as 3.5 × 4.5 mm^2^ to cover two melanoma spots under the skin, as shown by the white box region in Figure 4d (animal protocols were approved by the Institutional Animal Care and Use Committee, Pennsylvania State University, University Park, State College, PA, USA). 

Pulse energy after the spatial filter was set at ~600 nJ to yield an optical fluence of ~14.7 mJ/cm^2^, which was below the American National Standards Institute (ANSI) safety skin maximum permissible exposure (MPE) limit of 20 mJ/cm^2^ at 532 nm [32]. A-lines were averaged 300 times to generate the MAP image, as seen in Figure 4e. The image was then color-coded with a distance relative to the skin surface, which showed clear melanoma boundaries and depth information, as seen in Figure 4f. The feasibility of the high-contrast melanoma imaging demonstrated here could benefit clinical point-of-care depth detection and the monitoring of melanoma cells using wearable TUT-based OR-PAM.

The main limitations of our first-generation TUT-based OR-PAM system were scan speed (100 × 100 steps took 50 min) and SNR. Scan speed could be improved by using state-of-the-art scanning methods, such as galvo-mirror-based scanning of the optical beam like that employed in conventional OR-PAM systems. This could achieve a scanning speed of 1000 × 1000 steps in 100 s [7]. The SNR could be improved by further investigating novel high-acoustic absorption backing and proper impedance-matching layers. Using a better backing layer and the described two-matching-layer design, we could improve bandwidth by 60% [22,33] and transmission coefficient by ~160%. 

Furthermore, fabricating the TUT with a higher-frequency LiNbO_3_ wafer would further increase bandwidth [34] and lead to an improvement in the spatial resolution of the OR-PAM system. During the scanning, blood diffusion from certain areas of these *ex ovo* samples resulted in low-contrast vascular images: vascular contrast is expected to improve when imaging living subjects *in vivo*. Despite these limitations, the novel TUT-based OR-PAM approach presented here showed promising results that could lead to the miniaturization of OR-PAM for emerging wearable and high-throughput imaging applications. Additionally, the total cost of the proposed TUT is less than 50 USD, well below that of current OR-PAM setups that require additional optical components and instruments.

## 4. Conclusions

A novel OR-PAM system based on a transparent LiNbO_3_ ultrasound transducer was developed, characterized, and validated using both inanimate and biological subjects. The transducer was ITO-coated with 80% optical transmission in the visible and near-infrared optical-wavelength regions, and had a center frequency of 13 MHz with a fractional photoacoustic bandwidth of 60%. The resultant transparency of the LiNbO_3_ transducer facilitated a shared pathway for both light and acoustic-wave propagation. 

Our approach also removed the need for additional optical components (such as acoustic-optic prisms) and large-coupling media used in conventional OR-PAM systems. Instead, the OR-PAM presented in this work has a much smaller, lighter imaging head—the TUT itself—with minimal acoustic coupling. Imaging experiments demonstrated an SNR of 38 dB, and a lateral and axial resolution of 8.5 and 150 µm, respectively. 

The OR-PAM’s feasibility of vascular imaging was demonstrated using Day 4 CAM and melanoma depth profiling using melanoma-tissue phantoms. In the future, the proposed TUT could be integrated in conventional optical-microscopy techniques to allow multimodal microscopy that is capable of both ultrasound stimulation and sensing. Eventually, the TUT technology could be further scaled to develop miniaturized photoacoustic endomicroscopy and microendoscopy devices for space-constrained point-of-care clinical applications, e.g., prostate and pancreas needle biopsies [35,36,37].
